# Sex hormone changes in a 24-mo dietary and physical activity randomized intervention trial in postmenopausal females: the Diet, physical Activity and Mammography study (DAMA) study

**DOI:** 10.1016/j.ajcnut.2025.07.025

**Published:** 2025-07-31

**Authors:** Benedetta Bendinelli, Giovanna Danza, Melania Assedi, Fabio Villanelli, Giulia Vagnoni, Elisa Pastore, Ilaria Ermini, Calogero Saieva, Saverio Caini, Sara Marchiani, Linda Vignozzi, Giovanna Masala

**Affiliations:** 1 Clinical Epidemiology Unit, Institute for Cancer Research, Prevention and Clinical Network (ISPRO), Florence, Italy; 2 Department of Experimental Clinical and Biomedical Sciences "Mario Serio," University of Florence, Florence, Italy; 3 Cancer Risk Factors and Lifestyle Epidemiology Unit, Institute for Cancer Research, Prevention and Clinical Network (ISPRO), Florence, Italy; 4 Centro Regionale di Coordinamento Salute e Medicina di Genere (CISMEG), Careggi University Hospital, Florence, Italy

**Keywords:** Sex hormones, SHBG, diet, physical activity, randomized intervention trial, postmenopausal females

## Abstract

**Background:**

The role of sex steroid hormones in the etiology of several diseases, including breast and endometrial cancer, has been widely documented. In postmenopausal females, higher concentrations of estrogens and androgens are associated with a higher risk of developing breast cancer. It is therefore important to investigate whether and how diet and physical activity (PA) can modulate sex hormone blood concentrations.

**Objectives:**

We evaluated the effect of a 24-mo dietary and/or PA intervention on plasma concentrations of a series of sex hormones.

**Methods:**

The 234 study participants were healthy postmenopausal females aged 50–69 y, with high breast density, nonsmokers, and no hormone replacement therapy users. They were randomly assigned to the following 4 intervention arms: *1*) isocaloric dietary intervention, mainly plant-based; *2*) moderate-intensity exercise intervention with ≥1 h/wk of supervised strenuous activity; *3*) both dietary and exercise interventions; *4*) control group with general recommendations on healthy lifestyle. In plasma samples collected at baseline and at the end of the intervention, concentrations of estradiol, estrone, progesterone, 17-OH progesterone, testosterone, androstenedione, dehydroepiandrosterone, and dehydroepiandrosterone sulfate were determined by liquid chromatography tandem mass spectrometry methods. Sex hormone-binding globulin was determined by immunoassay, and free estradiol and testosterone were calculated using the Vermeulen method. Statistical analyses were performed using Tobit regression models.

**Results:**

After 24 mo, females randomly assigned to dietary treatment (arms 1 + 3) showed significant lower concentrations of estradiol [exp(*β*) 0.77; 95% confidence interval (CI): 0.61, 0.97; *P* value 0.03] and free estradiol (exp(*β*) 0.81; 95% CI: 0.65, 0.998; *P* value 0.048) compared with the control group (arms 2 + 4). No significant differences emerged for the other sex hormones. No effect of PA intervention was evident. Further adjustment for weight change that occurred during the intervention did not modify the results.

**Conclusions:**

Our results suggest that, in healthy postmenopausal females with high breast density, a healthy diet mainly based on plant food may play a role as a modulator of plasma estradiol concentration.

This trial was registered at the ISRCTN Registry (www.isrctn.com) as ISRCTN28492718.

## Introduction

During menopausal transition, with the decline of ovarian function, sex hormones stop having the synergistic and cyclic action aimed at maintaining reproductive function, and their blood concentrations along with their relative proportions also change [[1], [2], [3]]. The new balance is characterized by a sharp drop in estradiol concentrations and slight or no change in estrone concentrations, resulting in a reversal of the premenopausal estradiol:estrone ratio [1,2]. Estrone is, in fact, produced by peripheral tissues from androstenedione, whereas estradiol is produced by conversion of estrone [4]. It has been reported that in postmenopausal females, androstenedione aromatization occurs largely in adipose tissue, and a positive correlation between its concentrations and body weight has been shown. Furthermore, in post menopause, insufficient physical activity (PA) and obesity are correlated with increased estrogen and testosterone blood concentrations and with lower concentrations of sex hormone-binding globulin (SHBG), the protein that binds and transports sex steroid hormones, thus reducing their bioavailability [5].

Moreover, menopausal status, obesity, and different dietetic regimens are known to modulate gut microbiota composition. It is known that gut microbiota is an important regulator of circulating estrogen through the β-glucuronidase activity of specific bacterial species able to reactivate estrogens from their conjugated forms [[6], [7], [8]].

The role of sex steroid hormones in the etiology of several diseases, including breast and endometrial cancer, has been widely documented [[9], [10], [11]]. Several studies have shown a correlation between breast cancer (BC) risk and increased BMI (in kg/m^2^) in postmenopausal females, which correlates with an increase in bioavailable circulating estrogens [12,13]. Many of the risk factors for BC involve reproductive aspects, which suggests that sex hormones may play a role. Indeed, epidemiologic studies have shown that in postmenopause higher concentrations of estrogens and androgens are associated with a higher risk of developing BC [11]. In postmenopausal BC survivors, overweight and obesity have been associated with a worse prognosis [5,12,14].

Given the involvement of steroid hormones in the etiopathogenesis of several diseases, it is important to study whether and how lifestyle determinants can modulate their blood concentrations. Among lifestyle factors, cigarette smoking was positively correlated with androgen and estrogen concentrations [15]; regarding diet and PA, a recent review reported that studies involving interventions on both diet and exercise showed a decrease in estrogen concentrations. Some of the strongest associations included weight loss through diet and exercise interventions, reduction in alcohol intake, and consumption of a dietary pattern similar to the Mediterranean diet [16].

The Diet, physical Activity and Mammography study (DAMA) is a factorial randomized trial aimed at investigating whether a mainly plant-based diet and/or an increase in exercise were able to reduce mammographic breast density (BD) (primary outcome of the study) among postmenopausal females with elevated mammographic BD and therefore at increased risk of BC. The main results of the DAMA trial, i.e., the reducing effect of the dietary and the PA intervention on BD, were reported recently [17]. We then considered other secondary outcomes, namely blood concentrations of proinflammatory cytokines [18] and adipokines [19].

In the present study, we evaluated the potential effect of the DAMA trial on plasma concentrations of a series of steroid hormones, namely estradiol, estrone, progesterone, 17-OH progesterone, testosterone, androstenedione, dehydroepiandrosterone (DHEA), dehydroepiandrosterone sulfate (DHEAS), and SHBG. Some of these sex hormones have been indeed found to be particularly associated with PA and dietary regimen in postmenopause [[5], [6], [7], [8],^16^]. A specific high-sensitivity and semiautomatic liquid chromatography tandem mass spectrometry (LC-MS/MS) analysis method was set up for estrone and estradiol quantification in postmenopausal females.

This study was approved by the local Ethics Committee of the Tuscany Region.

## Methods

The DAMA study (trial registration number ISRCTN28492718, date of trial registration 17 May, 2012) is a single-center randomized intervention trial with a 2 × 2 factorial design*.* The DAMA study was approved by the Ethics Committee of the Local Health Authority “Azienda Sanitaria Firenze.” An informed consent was obtained from all study participants. The study protocol of the DAMA trial, including the objectives of the interventions and the performed activities, was reported in detail in a previous paper [20].

Participants were recruited between February 2009 and March 2010 among postmenopausal females attending the mammographic screening program implemented by Institute for Cancer Research, Prevention and Clinical Network (ISPRO) in the Florence area. Females with the following characteristics were recruited: age 50–69 y; high BD (BD > 50% according to the Breast Imaging Reporting and Data System classification [21]) assessed by a negative mammogram performed in the local screening program; no hormone therapy use in the past 12 mo; never smokers or former smokers since 6 mo or more; no previous diagnosis of cancer, diabetes, major cardiovascular and neurologic diseases or other diseases able to hamper their active participation in the study.

Recruited participants underwent a baseline visit with trained personnel who measured participants' weight, height, hip, and waist circumference and collected information on dietary and lifestyle habits through a validated food frequency questionnaire (FFQ) [22] and a standardized lifestyle questionnaire [23], both previously used in the European Prospective Investigation into Cancer and nutrition [24]. Data on dietary consumption, obtained through the FFQs, were transformed into estimates of nutrient intake according to specifically developed Italian Food Tables. The average dietary glycemic index (GI) and the individual glycemic load (GL) were also calculated for each subject. A detailed description of the entire procedure has been published elsewhere [25]. During the same baseline visit, the menopausal status of the study participants was ascertained through self-reported absence of menses for the past 12 mo.

A fasting venous blood sample was taken and, within 24 h after collection, processed into plasma, red cells, and buffy-coat aliquots and stored in the liquid nitrogen biological bank of the project.

After the baseline visit, participants were randomly assigned to 1 of the 4 arms of the study through permuted-block randomization stratified by age (50–59/60–69 y) and BMI category (<25/ ≥25), with a constant block size (*n* = 4). The 4 arms of the study were:

*Dietary intervention (arm #1)*: aimed to change the composition of diet toward greater consumption of vegetables, legumes, whole-grains, fish, and extravirgin olive oil, and lower consumption of red meat, milk, dairy products, desserts, and alcohol, in an isocaloric context and without specific advice on food quantity. Specific intervention objectives were: substitution of refined grains with whole-grains; consumption of both raw and cooked vegetables at each meal; high consumption of fish (2/3 times/wk), legumes and pulses (3/4 portions/wk) and fruit (3 portions/d); low consumption of red meat (<1 time/wk), cakes (1 portion/wk), milk or yogurt (1 portion/d), cheese (2 portions/wk), and wine (max 1 glass/d); extravirgin olive oil as the only dressing and cooking fat [17,20]. Participants were also invited to attend group meetings and cooking classes.

*PA intervention (arm #2)*: aimed to increase moderate daily recreational activities ≤1 h/d [walking or biking at moderate pace, ∼3 metabolic equivalent (MET)] and to perform ≥1 h/wk of more strenuous activity (6–10 MET) by attending specific sessions led by qualified personnel. Participants were also invited to attend group meetings and collective walks.

*Dietary + PA intervention (arm #3)*: combination of arms #1 and #2 activities. To reduce contamination, females in this arm had separate dietary and PA sessions from females randomly assigned to arms # 1 or # 2.

*Control arm (arm #4)*: general advice and printed material on dietary and PA recommendations on cancer prevention.

After the 24 mo (± 3 mo) of the DAMA trial intervention, participants underwent a final visit with the same procedure as the baseline visit.

### Plasma concentration measurement of steroid hormones and SHBG

Plasma samples collected at baseline and at the end of the intervention were retrieved from the study biological bank and shipped to the laboratory of the Department of Experimental, Clinical, and Biomedical Sciences, University of Florence, Italy, for the quantification of the steroid hormones and SHBG. To minimize the contribution of the analytical bias, baseline and follow-up samples for the same subject were always analyzed contemporaneously in the same analytical session.

Detailed laboratory methods for sex hormones and SHBG measurement in plasma samples are reported in Supplementary material. Briefly, the quantification of steroid hormones estradiol, estrone, progesterone, 17-OH progesterone, testosterone, androstenedione, DHEA, and DHEAS, was performed with 2 different isotopic dilution LC-MS/MS methods using a SCIEX 6500QTRAP (Biosystems/MDS SCIEX, Foster City, Calif/Concord) equipped with an Electrospray Ionization (ESI) source and coupled to an Agilent 1260 HPLC (Agilent). Sensitivity was evaluated by calculating the limit of detection and limit of quantification (LOQ). Lower LOQs were as follows: estradiol 5 pg/mL; estrone 9 pg/mL; progesterone 1.0 nmol/L; 17-OH progesterone 0.75 nmol/L; testosterone 0.6 nmol/L; androstenedione 0.5 nmol/L; DHEA 0.5 nmol/L; and DHEAS 64 nmol/L.

SHBG was determined with the Elecsys SHBG Cobas kit (Roche Diagnostics), an electrochemiluminescence immunoassay with a LOQ of 0.350 nmol/L.

### Statistical analyses

Sex hormones plasma concentrations falling below the LOQ were replaced with one-half the respective LOQ [26,27]. No samples had values above the upper LOQ. Sex hormones and SHBG mean plasma concentration and SDs were estimated at baseline and follow-up. Hormone concentration values were then log-transformed to normalize the distribution.

We applied Tobit regression models for left-censored variables [28,29] to evaluate the effect of the dietary and PA interventions on log-transformed sex hormones and SHBG concentrations at follow-up in comparison with the respective control groups. The 2 treatments were evaluated simultaneously in the same model and thus mutually adjusted. According to the factorial design of the DAMA trial, for the dietary intervention, we compared participants randomly assigned to arms #1 and #3 compared with arms #2 and #4. To evaluate the effect of the PA intervention, we compared participants randomly assigned to arms #2 and #3 compared with arms #1 and #4. The analyses were performed on an intention-to-treat basis. Regression models were adjusted for baseline log-transformed level of each hormone and randomization block variables (age and BMI) [30]. We also run the analyses, adding to the model the weight change (kg) that occurred during the intervention. Tobit regression coefficients (*β*), and relative 95% confidence intervals (CIs), were then back-transformed in the exponential form (exp(*β*)). An exp(*β*) value <1.0 indicates a lower hormone level in the treatment group than in the control group after the 24-mo intervention. Interaction between the 2 treatments was also tested [31].

Changes in selected foods and nutrients intake between baseline and the end of dietary treatment were calculated through a general linear model analysis adjusted for daily caloric intake.

The analyses were performed using Stata 14.1 statistical software (StataCorp LLC).

## Results

**FIGURE 1:**
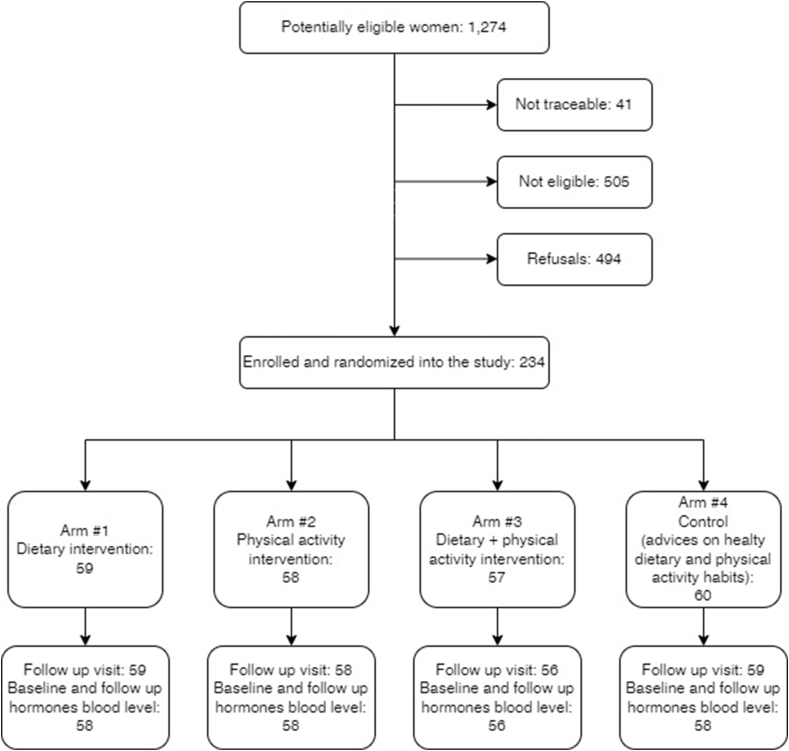
Participants’ recruitment, randomization, and follow-up in the DAMA factorial intervention trial. CONSORT flow diagram showing procedures of selection among eligible women and participants with complete data available. DAMA, Diet, Exercise and Mammography.

Selection procedures and final numbers of trial participants are reported in Figure 1. We recruited and randomly assigned a total of 234 females. This analysis included 230 participants with complete information and blood samples collected at baseline and at follow-up. The mean interval between the 2 visits was 2.0 y (SD 0.1).

The main characteristics of the study participants at baseline, overall, and by treatment are reported in Table 1. The mean age of participants was 58.6 y (SD 5.1), most participants were never smokers (57.8%), with BMI < 25.0 (63.5%), a high school degree (71.7%), and with 1 or more children (84.7%). Approximately 58% of participants had paid work that was mainly sedentary work (60.9%). No differences emerged between the treatment and control groups characteristics at baseline.

**TABLE 1:** Baseline distribution (categorical variables) or mean and SD (continuous variables) of selected characteristics of women enrolled into the Diet, Exercise and Mammography trial, overall and by treatment.

	Whole study sample (*n* = 230)	Dietary intervention		Physical activity intervention	
		Treatment group^1^ (*N* = 114)	Control group^2^ (*N* = 116)	Treatment group^3^ (*N* = 114)	Control group^4^ (*N* = 116)
General characteristics					
Age ( y)	58.6 (5.1)	58.6 (5.2)	58.6 (5.1)	58.7 (4.9)	58.6 (5.4)
Level of education (*n*)					
None/primary school	65 (28.3%)	30 (26.3%)	35 (30.2%)	28 (24.6%)	37 (31.9%)
High school	98 (42.6%)	51 (44.7%)	47 (40.5%)	50 (43.9%)	48 (41.4%)
University	67 (29.1%)	33 (29.0%)	34 (29.3%)	36 (31.6%)	31 (26.7%)
Smoking (*n*)					
Former	97 (42.2%)	53 (46.5%)	44 (37.9%)	52 (45.6%)	45 (38.8%)
Never	133 (57.8%)	61 (53.5%)	72 (62,1%)	62 (54.4%)	71 (61.2%)
Height (cm)	158.9 (6.0)	159.2 (5.8)	158.7 (6.3)	159.3 (5.3)	158.6 (6.7)
Weight (kg)	61.7 (9.4)	62.3 (9.5)	61.2 (9.4)	62.6 (10.1)	60.8 (8.7)
BMI (kg/m^2^)	24.4 (3.4)	24.5 (3.2)	24.3 (3.7)	24.6 (3.6)	24.2 (3.3)
BMI ≥ 25 (kg/m^2^)	84 (36.5%)	41 (36.0%)	43 (37.1%)	40 (35.1%)	44 (37.9%)
Waist circumference (cm)	76.5 (7.7)	76.6 (7.3)	76.4 (8.0)	76.8 (7.8)	76.2 (7.6)
Waist circumference ≥ 88 (cm)	17 (7.4%)	7 (6.1%)	10 (8.6%)	11 (9.6%)	6 (5.2%)
Women at work (*n*)	133 (57.8%)	68 (59.6%)	65 (56.0%)	66 (57.9%)	67 (57.8%)
Physical activity at work (*n*)					
Sedentary	81 (60.9%)	46 (67.7%)	35 (53.9%)	40 (60.6%)	41 (61.2%)
Standing	34 (25.6%)	13 (19.1%)	21 (32.3%)	17 (25.8%)	17 (25.4%)
Manual	18 (13.5%)	9 (13.2%)	9 (13.8%)	9 (13.6%)	9 (13.4%)
Total physical activity index^5^ (*n*)					
Inactive	59 (25.6%)	35 (30.7%)	24 (20.7%)	32 (28.1%)	27 (23.3%)
Moderate inactive	60 (26.1%)	27 (23.7%)	33 (28.4%)	30 (26.3%)	30 (25.9%)
Moderate active	92 (40.0%)	42 (36.8%)	50 (43.1%)	43 (37.7%)	49 (42.2%)
Active	19 (8.3%)	10 (8.8%)	9 (7.8%)	9 (7.9%)	10 (8.6%)
Hormonal and reproductive history					
Hormone therapy use (in the past, *n*)	72 (31.3%)	38 (33.3%)	34 (29.3%)	40 (35.1%)	32 (27.6%)
Contraceptive pill use (ever use, *n*)	126 (54.8%)	60 (52.6%)	66 (56.9%)	70 (61.4%)	56 (48.3%)
Number of children (*n*)					
(7 subjects with missing information)					
0	34 (15.2%)	19 (17.1%)	15 (13.4%)	19 (16.8%)	15 (13.6%)
1	93 (41.7%)	51 (45.9%)	42 (37.5%)	50 (44.2%)	43 (39.1%)
2+	96 (43.0%)	41 (36.9%)	55 (49.1%)	44 (38.9%)	52 (47.3%)
Lactation (ever, *n*)	152 (80.4%)	70 (76.1%)	82 (84.5%)	78 (83.0%)	74 (77.9%)
Age at first child (y)	27.7 (5.1)	27.6 (4.8)	27.8 (5.5)	27.5 (5.4)	27.9 (4.9)
Age at menarche (y)	12.6 (1.4)	12.5 (1.5)	12.7 (1.4)	12.4 (1.4)	12.7 (1.4)
Age at menopause (y)	49.9 (4.1)	49.9 (4.0)	49.9 (4.1)	49.1 (8.0)	49.4 (4.8)

1 Dietary + dietary and physical activity arms.

2 Physical activity + control arms.

3 Physical activity + dietary and physical activity arms.

4 Dietary + control arms.

5 Four levels based on a cross-tabulation between occupational and leisure time activity.

Overall, participants experienced an average weight loss of ∼0.34 kg (SD 0.003). The weight loss was more evident in the dietary + PA intervention arm (arm#3, −0.54 kg; SD 2.6) and less evident in the control arm (arm #4, −0.20 kg; SD 2.6).

Baseline and follow-up plasma concentrations of sex hormones and SHBG by treatment group are reported in Table 2 together with the results of the Tobit regression analysis. Five subjects with missing information for estradiol and estrone plasma concentrations and 3 subjects with missing information on SHBG plasma concentrations were excluded from the respective analysis. There were no significant differences in baseline hormone concentrations among the treatment group. For all sex hormones, excluding testosterone, DHEA, DHEAS, and SHBG, a general tendency to a reduction in the concentrations of the examined steroid hormones emerged at the end of the study period as compared with the respective baseline levels. The reduction ranged between 10% for estrone and 70% for estradiol and free estradiol. Results of the Tobit regression adjusted models showed a significantly lower follow-up level of estradiol (exp(*β*) 0.77; 95% CI: 0.61, 0.97; *P* value 0.03) and free estradiol (exp(*β*) 0.81; 95% CI: 0.65, 0.998; *P* value 0.048) in females randomly assigned to dietary intervention (arms #1 + #3) in comparison with females in the relative control group (arms #2 + #4). The same effect was not observed for any of the other hormones. No significant differences emerged in follow-up hormone concentrations in participants randomly assigned to PA intervention (arms #2 + #3) as compared with their control group (arms #1 + #4).

**TABLE 2:** Sex hormones and SHBG in the Diet, Exercise and Mammography trial: baseline and follow-up plasma concentrations and analysis of follow-up levels according to treatment group compared with control group.

	Dietary intervention			Physical activity intervention			*P* interaction between treatments
	Treatment group^1^*n* = 114	Control group^2^*n* = 116	*P* value^8^	Treatment group^3^*n* = 114	Control group^4^*n* = 116	*P* value^8^	
Estradiol (pg/mL) 5 subjects with missing information 27 subjects with Follow-up (FU) hormone concentration under LOD^5^							0.42
Mean at baseline (SD)	10.91 (34.57)	7.12 (18.92)	0.52	8.84 (25.32)	9.19 (30.31)	0.76	
Mean at follow-up (SD)	4.48 (7.37)	5.10 (7.48)		5.31 (9.79)	4.28 (3.80)		
Adjusted mean at follow-up (SD)^6^	3.06 (1.80)	3.82 (1.74)		3.44 (1.85)	3.43 (1.78)		
Exp(*β*) (95% CI)^7^	0.77 (0.61, 0.97)	Ref.		1.01 (0.80, 1.28)	Ref.		
*P* value	0.03			0.92			
Free estradiol (pg/mL) 8 subjects with missing information							0.39
Mean at baseline (SD)	0.29 (0.87)	0.20 (0.56)	0.71	0.25 (0.74)	0.24 (0.72)	0.64	
Mean at follow-up (SD)	0.12 (0.20)	0.13 (0.16)		0.14 (0.24)	0.11 (0.10)		
Adjusted mean at follow-up (SD)^6^	0.09 (0.05)	0.10 (0.05)		0.09 (0.05)	0.09 (0.05)		
Exp(*β*) (95% CI)^7^	0.81 (0.65, 0.998)	Ref.		1.05 (0.85, 1.29)	Ref.		
*P* value	0.048			0.67			
Estrone (pgm/L) 5 subjects with missing information 1 subject with FU hormone concentration under LOD^5^							0.17
Mean at baseline (SD)	24.46 (18.01)	24.38 (12.80)	0.70	24.95 (13.74)	24.90 (17.33)	0.51	
Mean at follow-up (SD)	23.59 (12.34)	24.28 (11.18)		23.35 (11.85)	24.52 (11.67)		
Adjusted mean at follow-up (SD)^6^	22.08 (5.84)	23.04 (5.24)		21.99 (4.93)	23.12 (6.09)		
Exp(*β*) (95% CI)^7^	0.95 (0.87, 1.04)	Ref.		0.94 (0.86, 1.03)	Ref.		
*P* value	0.28			0.20			
Testosterone (nmol/L) 7 subjects with FU hormone concentration under LOD^5^							0.61
Mean at baseline (SD)	0.69 (0.30)	0.72 (0.39)	0.59	0.69 (0.30)	0.72 (0.39)	0.75	
Mean at follow-up (SD)	0.75 (0.46)	0.74 (0.43)		0.74 (0.43)	0.75 (0.46)		
Adjusted mean at follow-up (SD)^6^	0.67 (0.28)	0.71 (0.37)		0.69 (0.29)	0.69 (0.37)		
Exp(*β*) (95% CI)^7^	0.98 (0.87, 1.11)	Ref.		1.05 (0.93, 1.18)	Ref.		
*P* value	0.77			0.44			
Free testosterone 3 subjects with missing information							0.73
Mean at baseline (SD)	0.012 (0.005)	0.013 (0.008)	0.86	0.012 (0.005)	0.013 (0.008)	0.54	
Mean at follow-up (SD)	0.013 (0.009)	0.013 (0.008)		0.013 (0.008)	0.013 (0.009)		
Adjusted mean at follow-up (SD)^6^	0.012 (0.005)	0.013 (0.007)		0.012 (0.005)	0.012 (0.007)		
Exp(*β*) (95% CI)^7^	0.97 (0.87, 1.10)	Ref.		1.07 (0.95, 1.21)	Ref.		
*P* value	0.66			0.24			
Testosterone/estradiol ratio 5 subjects with missing information							0.70
Mean at baseline (SD)	0.29 (0.37)	0.29 (0.41)	0.75	0.28 (0.36)	0.28 (0.42)	0.74	
Mean at follow-up (SD)	0.41 (0.78)	0.28 (0.43)		0.33 (0.52)	0.36 (0.72)		
Adjusted mean at follow-up (SD)^6^	0.25 (0.15)	0.20 (0.12)		0.23 (0.14)	0.22 (0.13)		
Exp(*β*) (95% CI)^7^	1.03 (0.98, 1.31)	Ref.		1.03 (0.89, 1.19	Ref.		
*P* value	0.08			0.67			
Androstenedione (nmol/L) 1 subject with FU hormone concentration under LOD^5^							0.99
Mean at baseline (SD)	1.89 (0.77)	1.78 (0.70)	0.27	1.87 (0.72)	1.81 (0.76)	0.29	
Mean at follow-up (SD)	1.74 (0.77)	1.82 (0.86)		1.72 (0.72)	1.84 (0.89)		
Adjusted mean at follow-up (SD)^6^	1.65 (0.47)	1.71 (0.48)		1.63 (0.45)	1.72 (0.49)		
Exp(*β*) (95% CI)^7^	0.92 (0.84, 1.01)	Ref.		0.93 (0.84, 1.02)	Ref.		
*P* value	0.10			0.10			
Progesterone (nmol/L) 164 subjects with FU hormone concentration under LOD^5^							0.32
Mean at baseline (SD)	0.21 (0.27)	0.23 (0.33)	0.54	0.20 (0.20)	0.24 (0.38)	0.54	
Mean at follow-up (SD)	0.19 (0.23)	0.19 (0.25)		0.19 (0.23)	0.19 (0.26)		
Adjusted mean at follow-up (SD)^6^	0.07 (0.11)	0.07 (0.11)		0.06 (0.079)	0.07 (0.14)		
Exp(*β*) (95% CI)^7^	1.14 (0.71, 1.84)	Ref.		1.08 (0.67, 1.74)	Ref.		
*P* value	0.59			0.76			
17OH-progesterone (nmol/L) 15 subjects with FU hormone concentration under LOD^5^							0.91
Mean at baseline (SD)	1.03 (0.61)	0.93 (0.20)	0.22	0.96 (0.48)	1.00 (0.62)	0.99	
Mean at follow-up (SD)	0.94 (1.02)	0.90 (0.48)		0.85 (0.47)	0.99 (1.01)		
Adjusted mean at follow-up (SD)^6^	0.77 (0.29)	0.79 (0.27)		0.75 (0.25)	0.80 (0.30)		
Exp(*β*) (95% CI)^7^	0.92 (0.78, 1.09)	Ref.		0.96 (0.81, 1.13)	Ref.		
*P* value	0.10			0.10			
DHEA (nmol/L) 2 subjects with FU hormone concentration under LOD^5^							0.94
Mean at baseline (SD)	20.73 (11.62)	21.64 (11.03)	0.45	21.66 (11.70)	20.72 (10.95)	0.65	
Mean at follow-up (SD)	19.80 (12.25)	20.57 (10.98)		20.33 (12.21)	20.05 (11.04)		
Adjusted mean at follow-up (SD)^6^	19.19 (10.21)	20.13 (9.72)		19.71 (10.11)	19.62 (9.85)		
Exp(*β*) (95% CI)^7^	0.99 (0.94, 1.05)	Ref.		0.96 (0.91, 1.02)	Ref.		
*P* value	0.83			0.21			
DHEAS (nmol/L) 1 subject with FU hormone concentration under LOD^5^							0.46
Mean at baseline (SD)	1722.83 (882.91)	1846.30 (986.78)	0.38	1820.69 (969.92)	1750.13 (905.79)	0.66	
Mean at follow-up (SD)	1673.48 (987.60)	1766.35 (942.83)		1728.19 (973.03)	1712.57 (959.78)		
Adjusted mean at follow-up (SD)^6^	1628.28 (779.13)	1736.90 (865.59)		1688.55 (837.97)	1677.67 (813.38)		
Exp(*β*) (95% CI)^7^	0.99 (0.95, 1.05)	Ref.		0.97 (0.92, 1.02)	Ref.		
*P* value	0.95			0.24			
SHBG (ngm/L) 3 subjects with missing information; 0 subject with FU hormone concentration under LOD^5^							0.36
Mean at baseline (SD)	33.80 (14.07)	36.16 (13.63)	0.09	35.21 (13.77)	34.77 (14.04)	0.72	
Mean at follow-up (SD)	34.90 (12.74)	36.36 (14.11)		35.18 (13.10)	36.08 (13.80)		
Adjusted mean at follow-up (SD)^6^	34.64 (11.89)	35.60 (11.34)		34.75 (11.23)	35.50 (11.99)		
Exp(*β*) (95% CI)^7^	1.03 (0.98, 1.08)	Ref.		0.97 (0.93, 1.01)	Ref.		
*P* value	0.21			0.16			

Abbreviations: CI, confidence interval; DHEA, dehydroepiandrosterone; DHEAS, dehydroepiandrosterone sulfate; LOD, limit of detection; SHBG, sex hormone-binding globulin.

1 Dietary + dietary and physical activity arms.

2 Physical activity + control arms.

3 Physical activity + dietary and physical activity arms.

4 Dietary + control arms.

5 Plasma hormone concentrations falling below the LOD were replaced with LOD/2.

6 Adjusted for log-transformed hormone level at baseline and randomization block variables (age and BMI).

7 Tobit model adjusted for baseline log-transformed hormone level and randomization block variables (age and BMI).

8 *P* values according to treatments from Kruskal–Wallis equality-of-populations rank test.

Results of analyses further adjusted for weight change that occurred in the 24 mo of the intervention were materially unchanged (data not shown).

No interaction effect emerged between dietary and PA treatments for any steroid hormone or SHBG.

Baseline consumptions of selected food and nutrients and their changes at the end of the dietary intervention are shown in Table 3. A significant increase in consumption of vegetables (21.1%), legumes (83.2%), and whole grain bread (172.3%), and a decrease in consumption of red meat (−61.2%) and cakes (−31.7%) emerged in the dietary treatment group in comparison with the control group. Changes in nutrient intake were also evident with a significant increase in fiber (17.8%) and a reduction in animal protein (−18.6%) and in the vegetable protein/animal protein ratio (−18.6%). A slight but significant increase in GI (2.5%) emerged in the dietary treatment group in comparison with the control group, whereas a 9.3% not significant reduction in GL was also observed in comparison with the control group, where a 3.1% reduction was found.

**TABLE 3:** Baseline consumption of selected food and nutrients, absolute change, and relative percent change in consumption at the end of the dietary intervention in the Diet, Exercise and Mammography trial.

	Dietary treatment group^1^*n* = 114		Control group^2^*n* = 116		*P* value^3^
	Baseline mean consumption (SD)	Absolute change (%)	Baseline mean consumption (SD)	Absolute change (%)	
Food group (g/d)					
Total vegetables	200.5 (76.8)	42.3 (21.1)	199.7 (109.0)	−3.9 (1.9)	<0.0001
Leafy vegetables	35.0 (20.5)	11.8 (33.7)	36.0 (24.4)	3.2 (8.9)	0.009
Fresh fruit	321.7 (160.9)	−1.8 (0.6)	343.7 (182.6)	4.1 (1.2)	0.35
Nuts, seed, and dried fruit	2.5 (4.7)	2.4 (96.0)	2.6 (3.7)	0.8 (30.8)	0.02
Legumes	20.8 (17.1)	17.3 (83.2)	17.8 (11.9)	3.1 (17.4)	<0.0001
Red and processed meat	71.7 (39.1)	−43.9 (61.2)	73.2 (45.8)	−22.1 (30.2)	<0.0001
Cheese	47.8 (35.2)	−17.2 (36.0)	42.9 (32.3)	−8.3 (19.3)	0.06
White bread	77.5 (77.1)	−38.0 (49.0)	78.1 (67.6)	−21.6 (27.6)	0.13
Whole grain bread	22.4 (38.7)	38.6 (172.3)	20.6 (31.4)	10.1 (49.0)	0.0005
Cakes and cookies	69.3 (74.0)	−22.0 (31.7)	60.0 (44.9)	6.6 (11.0)	0.005
Wine	72.4 (99.1)	−16.7 (23.1)	71.8 (101.0)	−2.0 (2.8)	0.02
Extravirgin olive oil	27.6 (10.9)	4.4 (15.9)	28.2 (14.4)	−0.7 (2.5)	0.002
Nutrients					
Fiber **(**g/d)	21.4 (7.1)	3.8 (17.8)	21.5 (7.3)	0.3 (1.4)	0.0001
Animal protein (g/d)	55.3 (18.7)	−10.3 (18.6)	55.1 (20.0)	−0.8 (1.4)	0.0001
Vegetable protein (g/d)	27.9 (10.4)	0.1 (0.3)	28.2 (10.0)	−1.3 (4.6)	0.24
Veg protein/animal protein ratio	2.1 (0.8)	−0.4 (18.6)	2.1 (0.8)	0.04 (1.9)	0.0003
Animal fat (g/d)	42.9 (18.6)	−12.0 (28.0)	40.6 (16.6)	−1.2 (2.9)	<0.0001
Vegetable fat (g/d)	38.2 (13.6)	5.2 (13.6)	38.3 (16.2)	−0.2 (0.5)	0.004
Carbohydrates (g/d)	255.8 (96.0)	−29.9 (11.7)	258.9 (86.4)	−7.8 (3.0)	0.04
Glycemic index	53.7 (2.9)	1.35 (2.5)	54.0 (2.7)	−0.14 (0.3)	0.003
Glycemic load	137.9 (54.0)	−12.9 (9.3)	140.0 (47.6)	−4.4 (3.1)	0.18

1 Dietary + dietary and physical activity arms.

2 Physical activity + control arms.

3 *P* values calculated from general linear model (adjusted for daily caloric intake) comparing the dietary treatment group with the control group.

## Discussion

In this secondary analysis of the DAMA trial carried out in healthy postmenopausal females, we observed that, at the end of the 24-mo intervention, participants randomly assigned to the dietary treatment showed lower plasma concentrations of total estradiol and free estradiol in comparison with the control group, whereas no significant effect emerged for the other hormones. No effect of the PA intervention on steroid hormone concentrations emerged.

Several trials previously evaluated the association between dietary factors and sex hormone concentrations among postmenopausal females with not entirely consistent results. In the trial by Kaaks et al. [32], the dietary intervention, aimed at the reduction of total fat and refined carbohydrates intake and an increase in foods rich in dietary fiber and phytoestrogens, was not associated with estradiol or free estradiol concentrations reduction. Brown et al. [33] found no association between sex steroid hormone concentrations and diet (not even adding PA) in postmenopausal females, overweight or with obesity, and physically inactive, who had survived BC. Finally, in a trial aimed to assess the effect of Mediterranean diet on the profile of endogenous estrogens in healthy postmenopausal females, a significant decrease of total urinary estrogen concentrations emerged among participants in the intervention group [34].

Notably, a direct association between estrogen concentrations and diets characterized by a Western-type pattern of high fat and red meat consumption emerged in cross-sectional studies [35,36].

Other studies among females with obesity or overweight have found an inverse association between intentional weight loss, as a consequence of low-fat diet or exercise interventions, and the concentration of some sex hormones. In the trial by Stolzenberg-Solomon et al. [37], the weight loss, driven by the increase of PA and the adherence to the Dietary Approaches to Stop Hypertension diet (high in fiber and low in fat content), resulted associated with a decrease in estrone, estradiol, free estradiol, and free testosterone concentrations. In the more recent trial by Duggan et al. [38], participants who maintained weight loss at 30 mo after the intervention (reduced calories diet and exercise) had a greater decrease in free estradiol and free testosterone.

Among the most investigated aspects in the literature regarding the association between diet, sex hormones, and the risk of BC, there is fiber intake [[39], [40], [41], [42], [43], [44], [45]]**.** In the DAMA trial, females randomly assigned to the dietary intervention were asked to gradually adopt a diet mainly based on plant foods, with a high intake of fiber, and a reduction in animal fats, and consequently with a low GL, low saturated- and trans-fats, and alcohol, and rich in antioxidants.

Our analysis has been conducted on intention-to-treat basis; however, participants in the DAMA trial actually experienced good compliance with the interventions, as shown by changes in dietary and PA habits in agreement with the objective of the assigned treatment. In particular, participants randomly assigned to the dietary intervention reported, at the end of the study, an increase in the consumption of total vegetables, nuts and seeds, legumes, and extravirgin olive oil, and a decrease in the consumption of red meat. The dietary intervention, thus, led to an increase in fiber intake and a consequent reduction in GL. The slight increase in GI that we observed in the dietary treatment group in comparison with the control group was probably driven by the strong reduction in red and processed meat (GI = 0) and its substitution with legumes and vegetables.

Steroid hormones circulate in the bloodstream bound to plasma proteins or free. The free fraction is the most bioavailable [46]. It is the liver's job to decrease the free fraction of circulating hormones or to deactivate them through various hydroxylation, methylation, and glucuronidation reactions that facilitate their excretion. Deactivated hormones pass through the bile to the intestine, where the gut microbiota, and in particular the estrobolome, can reactivate them. The estrobolome is a set of bacterial genes expressing beta-glucuronidase, an enzyme that deconjugates estrogens and converts them to their active form. Liver and intestine can thus repeatedly pass hormones to each other in a process referred as enterohepatic circulation, contributing to calibrate circulating hormone concentrations or their excretion [[46], [47], [48]]. Dietary interventions can modulate this process through ≥2 different mechanisms. Dietary fiber, by binding estrogens, can speed up their intestinal transit and elimination [38,49,50] playing a crucial role in reducing the residence time of deactivated estrogen compounds in the intestine [51], thus reducing the possibility of their deconjugation and reabsorption. The other mechanism resides in the modulation of gut microbiota composition with a lowering of gut bacterial β-glucuronidase activity and the consequent reduction of deconjugation and reabsorption of estrogens. This modulation increases the excretion of conjugated estrogen compounds, leading to a reduction in blood estrogen concentrations [49,50]. Recently, the estrobolome has received increasing attention, and microbiota β-glucorinidase activity has been correlated to the pathogenesis and progression of BC and has been identified as a possible therapeutic target [8,52].

The binding of estrogens to fiber but, above all, the modulation of the estrabolome with a decrease in β-glucuronidase activity following the dietary intervention could be a possible explanation for the results of our study. Notably, we observed a significant reduction in the plasma concentration of estradiol but not in the estrone plasma concentration. Estradiol is, in fact, more extensively metabolized by β-glucorinidase than estrone [53].

In our study, no effect on plasma hormone concentrations emerged in females randomly assigned to the PA intervention. However, in observational studies, total PA was in general negatively associated with concentrations of estrone, estradiol [5,[54], [55], [56]], androstenedione [5]**,** and testosterone [55], and positively correlated with SHGB [55,56]. This effect was also observed in 2 randomized controlled trials among sedentary postmenopausal females, the “Alberta Physical Activity and Breast Cancer Prevention Trial” [57] and the previous trial from McTiernan et al. [58], where the participants randomly assigned to the 12 mo interventions had statistically significant decreases in blood concentrations of estradiol, and free estradiol. In the latter study, involving females with obesity or overweight, the effect was significant also for estrone, but the associations were limited to subjects who experienced a decrease in BMI, suggesting an involvement of adipose tissue aromatase activity. The lack of effect observed in our trial could be due to the kind of PA treatment proposed to our participants which seems to be a milder one compared with interventions applied in the previously mentioned trials. On the other hand, as reported in previous papers, exercise interventions may not have a large impact on overall body weight loss but can affect body composition, and changes in fat mass may be correlated with changes in sex steroid hormones, particularly in postmenopausal females [59]. The lack of body composition and body fat distribution measures represents, in this sense, a limitation of our study.

Moreover, subjects in the DAMA trial were mainly normal-weight participants, a condition that may have undermined some of the potential benefits of the interventions on sex steroid hormones via weight loss.

Notably, the DAMA trial offered the opportunity to evaluate the effect of modifications of dietary and PA habits on healthy females characterized by a high risk of BC related to high BD and not selected on the basis of body weight as in many of the previously reported studies.

Participants in the DAMA trial, being healthy females not affected by metabolic syndrome and mainly not overweight or obese, had not extreme mean values of circulating steroid hormones at baseline, as shown by the proportion of hormone values below the LOQ.

Steroids determination in biological fluids has always been challenging and immunoassays, which are still the most diffused methods for their quantification in clinical laboratories, may present cross-reactivity problems that seriously affect the accuracy of the results, especially for sex steroids (e.g., testosterone and estradiol) at low concentrations. For these reasons, we used LC-MS/MS, considered the reference standard technique, for steroids quantification, and we applied Tobit regression models to estimate the regression parameters, overcoming the issue of hormone concentrations below the LOQ. Simulation studies showed that Tobit regression is substantially unbiased with log-normally distributed data and a percent of LOQ <50%.

Another strength of the DAMA study is represented by an approach aimed at achieving a general change in diet and/or PA habits in daily life for a relatively long period compared with most of the intervention studies reported in the literature.

Thus, in conclusion, our results obtained in an intervention trial suggest that a diet mainly based on plant food, with a low GL, low in saturated fats and alcohol, and rich in antioxidants and fiber, might play a role in the modulation of plasma estrogen concentration in postmenopausal females. Considering the role of sex steroid hormones in the etiology of BC [9,11], our study confirms the importance of the primary prevention activities aimed at improving lifestyle habits to prevent the carcinogenic processes.

## Author contributions

The authors’ responsibilities were as follows – GM: designed the research; GD, FV, LV, IE: conducted research; MA, BB, EP: analyzed data or performed statistical analysis; BB, GM: wrote paper; GM, BB, CS, SC, LV, GD, EP: primary responsibility for final content; and all authors: read and approved the final manuscript.

## Data availability

Data described in the manuscript, code book, and analytic code will be made available on request.

## Funding

The research leading to these results has received funding from the Associazione Italiana per la Ricerca contro il Cancro (AIRC) IG 2019, ID 23702, P.I. Giovanna Masala. The supporting source had no involvement or restrictions regarding publication.

## Conflict of interest

All authors report no conflicts of interest.
