# Supplementary material for: Sex hormone changes in a 24-mo dietary and physical activity randomized intervention trial in postmenopausal females: the Diet, physical Activity and Mammography study (DAMA) study
Source: Am J Clin Nutr. 2025 Jul 31;122(4):1111–20. doi: 10.1016/j.ajcnut.2025.07.025 (PMC12674079; doi:10.1016/j.ajcnut.2025.07.025)
Supplement: Multimedia component 1 [file mmc1.doc]

**Sex hormone changes in a 24-month dietary and physical activity randomized intervention trial in postmenopausal women: the DAMA study**

Benedetta Bendinelli

**Supplementary material**

**Laboratory methods for plasma level measurement of steroid hormones and sex hormones binding protein (SHBG)**

Plasma samples collected at baseline and at the end of the intervention were retrieved from the study biological bank and shipped to the laboratory of the Department of Experimental, Clinical, and Biomedical Sciences, University of Florence, Italy, for the quantification of the steroid hormones and SHBG. To minimize the contribute of the analytical bias, baseline and follow up samples for the same subject were always analyzed contemporaneously in the same analytical session. The quantification of steroid hormones estradiol, estrone, progesterone, 17-OH progesterone, testosterone, androstenedione, dehydroepiandrosterone (DHEA), dehydroepiandrosterone sulphate (DHEAS), dihydrotestosterone (DHT), was performed with two different isotopic dilution liquid chromatography tandem mass spectrometry methods (ID LC-MS/MS) using a SCIEX 6500QTRAP (Biosystems/MDS SCIEX, Foster City, Calif/Concord, Ontario, Canada) equipped with an ESI source and coupled to an Agilent 1260 HPLC (Agilent, Santa Clara, CA, US).

Estradiol and estrone were extracted using a Tecan Resolvex A200 96 well plates automated extraction system. The extraction was performed following the instructions of a technical note from Sciex^1^ with some modifications. SPE columns NBE Cerex Maestro A 1ml/ 5mg in the 96 well format were used. Serum (250 μL) was diluted with 500 μL of H_2_O/Isopropanol (95/5, v/v) and 50 μL of the internal standards solution (estradiol -D_4_ 500 pg/mL and estrone -D_3_ 500 pg/mL in MeOH) were added. The SPE columns were conditioned with CH_3_OH (300 μL) and H_2_O (300 μL) and the diluted samples were loaded. The columns were washed with H_2_O/Isopropanol 95/5 (300 μL) and dried for 8 minutes to eliminate the water, using the “Dry” function of the instrument, then the SPE were washed with Hexane (250 μL), and dried again for 1 minute. Estrogens were eluted with 150 μL x 2 of a mixture of Hexane/Ethylacetate (80/20) into a 96 deep well plate, and the eluate was dried for 30 minutes at room temperature. The dried extract was redissolved in 150 μL of H2O/MeOH 70/30 and stirred in an orbital shaker for 10 minutes, then 50 μL were injected.

The chromatographic system consisted of a clean-up/concentrating column (C18 Luna 5μm, 20x2mm) and an analytical column (PFP Luna 3μm, 50x2mm). The loading phase was H_2_O/CH_3_OH/CH_3_CN 80:10:10 v/v (0.5 mL /min for 4 min) while the eluents for the analytical separation were: Eluent A: H_2_O + NH_4_F 0.2 mM and Eluent B: CH_3_OH. The gradient separation program was 50% A (for 4 min) and then to 5% A in 4.5 min (flow 0.35 mL/min). Data were acquired in negative ion mode and selected transitions in MRM experiments were respectively: estradiol: 271>145 (quantifier), 271>183 (qualifier); estrone: 269>183 (quantifier), 269>145 (qualifier); estradiol-D_3_: 274>145; estrone-D_4_: 273>147. For each analytical session the calibration curve (Tecan Steroid panel LC-MS caibrators, blank + six points, range 10-1280 pg/mL for estrone and 30-3850 pg/mL for estradiol) was injected in triplicate and quantitative analysis was performed using the software MULTIQUANT (SCIEX).

All the other steroids (testosterone, androstenedione, progesterone, 17-OH progesterone, DHT, DHEA and DHEAS) were quantified by a laboratory developed method here briefly described. Serum 50 μL was deproteinized adding 400 μL of CH_3_CN + 0.1% formic acid containing the internal standards (Androstene-3,17-dione-2,3,4-^13^C_3_, 0.625 ng/mL; Testosterone-2,3,4-^13^C_3_, 0.625 ng/mL; 5α-Dihydrotestosterone-16,16,17-D_3_, 0.4 ng/mL; Progesterone-2,3,4-^13^C_3_, 1.25 ng/mL; 17α-Hydroxyprogesterone-2,3,4-^13^C_3_, 0.125 ng/mL; DHEA-2,2,3,4,4,6-D_6_, 4 ng/mL; DHEAS-2,2,3,4,4,6-D_6_, 500 ng/mL). After mixing and centrifugation the supernatant was transferred in a glass autosampler vial and diluted with 550 μL of H_2_O for the injection (25 μL). The chromatographic system consisted of a clean-up/concentrating column (C18 Luna 5μm, 20x2mm) and an analytical column (C18 Luna 3μm, 30x2mm). The loading phase was H_2_O/CH_3_OH 95:5 v/v (1.0 mL /min for 2,6 min) while the eluents for the analytical separation were: Eluent A: H_2_O + formic acid 10 mM / 5 mM NH_4_ formate and Eluent B: CH_3_OH + formic acid 10 mM / 5 mM NH_4_ formate. The gradient separation program was 50% A (for 2.5 min) and then to 20% A in 1.5 min, than to 5% A in 0.25 min (flow 0.30 mL/min). Data were acquired in negative ion mode for DHEAS and in positive ion mode for all the other steroids and selected transitions in MRM experiments were respectively: androstenedione 287>97 (quantifier), 287>109 (qualifier), androstenedione-^13^C_3_ 290>100; testosterone 289>109 (quantifier), 289>97 (qualifier), testosterone-^13^C_3_ 292>97; DHT 291>255 (quantifier), 291>159 (qualifier), DHT-D_3_ 294>258; progesterone 315>97 (quantifier), 315>109 (qualifier), progesterone-^13^C_3_ 318>100; 17-OH progesterone 331>97 (quantifier), 331>109 (qualifier), 17-OH progesterone-^13^C_3_ 334>100; DHEA 271>213 (quantifier), 271>253 (qualifier), DHEA-D_6_ 277>219; DHEAS 367>97 (quantifier), DHEAS-D_6_ 373>97. For each analytical session the calibration curve (6PLUS1® Multilevel Serum Calibrator Set Steroid Panel 1, Chromsystems, androstenedione (0.71-46.80 nM); testosterone (0.16-39.70 nM); DHT (0.18-4.66 nM); progesterone (0.44-76.0 nM); 17-OH progesterone (0.32-66.70 nM); DHEA (3.5-189.0 nM); DHEAS (323.0-15400 nM)) was injected in triplicate and quantitative analysis was performed using the software MULTIQUANT (SCIEX). Accuracy, intra-assay and inter-assay precision of the methods were evalued analysing commercial Quality Controls (MassCheck® Steroid Panel 1 Serum Controls, Chromsystems level I, level II, level III and Tecan Steroid panel LC-MS controls level I, level II) in pentaplicate in five different experiments performed in different days. CV% intra–assay was below 15% and CV% inter-assay below 20% for all the steroids analysed. Accuracy was in the range 80-120%. Sensitivity was evalued calculating limit of detection (LOD) and limit of quantification (LOQ) using the linear regression analysis on the first four points of calibration curves and the following formulas LOD = 3.3 x SE(intercept)/slope, LOQ = 10 x SE(intercept)/slope. Lower limits of quantification (LOQ) were as follows: progesterone 1.0 nmol/L; 17-OH progesterone 0.75 nmol/L; testosterone 0.6 nmol/L; androstenedione 0.5 nmol/L; DHEA 0.5 nmol/L; DHEAS 64 nmol/L; DHT 0.13 nmol/L. To evaluate the LOD and LOQ for E2 and E1, a specific calibration curve in a low concentration range was used (5-200 pg/mL for E2 and 10-400 pg/mL for E1). Lower limits of quantification (LOQ) were as follows: estradiol 5 pg/mL; estrone 9 pg/mL.

SHBG was determined with the Elecsys SHBG Cobas kit (Roche Diagnostics), an electrochemiluminescence immunoassay with a limit of detection of 0.350 nmol/L.

^1^ https://sciex.com/content/dam/SCIEX/pdf/tech-notes/all/Measurement_of_a_Panel_of_Steroids_by_LC-MS_MS__Employing_Rapid_Polarity_Switching_Poster.pdf (last accessed 8/10/2024)
